# Feasibility and Effectiveness of a Peer Referral Incentive Intervention to Promote Male Circumcision Uptake in Zambia

**DOI:** 10.1097/QAI.0000000000000808

**Published:** 2016-10-06

**Authors:** Arianna Zanolini, Carolyn Bolton, Lane-Lee Lyabola, Gabriel Phiri, Alick Samona, Albert Kaonga, Harsha Thirumurthy

**Affiliations:** * Centre for Infectious Disease Research in Zambia, Lusaka, Zambia;; † Department of Medicine, University of North Carolina at Chapel Hill, Chapel Hill, NC;; ‡ Society for Family Health, Lusaka, Zambia; and; § Ministry of Community Development, Mother and Child Health, Lusaka, Zambia.

**Keywords:** male circumcision, demand creation, peer referrals, economic incentives

## Abstract

Medical male circumcision is a promising HIV prevention tool in countries with generalized HIV epidemics, but demand creation interventions are needed to support scale-up. We piloted a peer referral intervention in which circumcision clients were offered incentives for referring their peers for circumcision.

**Methods:**

The intervention was implemented between June 2014 and February 2015 in 6 randomly selected health facilities in Southern Province, Zambia. For the first 5 months, circumcision clients ≥18 years of age were given referral vouchers that allowed them to refer up to 5 peers for circumcision within a 3-month period. An incentive of US$2 was offered for each referral. The primary outcome was the number of circumcisions performed per month in each facility. To assess the effect of the intervention, a difference-in-difference analysis was performed using longitudinal data from the intervention facilities and 22 nonintervention facilities. A questionnaire was also implemented to understand men's perceptions of the intervention.

**Results:**

During the 8-month intervention period, 1222 men over 18 years of age were circumcised in intervention facilities. In the first 5 months, 699 circumcision clients were enrolled and 385 clients brought a referral voucher given to them by an enrolled client. Difference-in-difference analyses did not show a significant increase in circumcisions performed in intervention facilities. However, circumcision clients reported that the referral incentive motivated them to encourage their friends to seek male circumcision. Peer referrals were also reported to be an important factor in men's decisions because 78% of clients who were referred reported that talking with a circumcised friend was important for their decision to get circumcised.

**Conclusions:**

The peer referral incentive intervention for male circumcision was feasible and acceptable. However, the intervention did not have a significant effect on demand for male circumcision. Barriers to circumcision and features of the intervention may have limited the effect of the intervention. Further efforts regarding encouraging male-to-male communication and evaluations with larger sample sizes are needed.

## INTRODUCTION

Medical male circumcision has been shown to reduce men's risk of acquiring HIV by up to 60%,^1–3^ and subsequently has been recognized as an essential tool for HIV prevention in high HIV prevalence countries.^4^ In Zambia, a major scale-up of voluntary medical male circumcision (VMMC) services has occurred in the past 5 years. Between 2007 and 2014, more than 850,000 circumcisions were performed, with more than 500,000 of them occurring in 2013 and 2014. However, prevalence of MC remains low at 22% and well below the country's target of 80%.^5,6^ As in several other countries in eastern and southern Africa, novel demand creation interventions are needed to achieve higher circumcision prevalence.^7^

Interpersonal communication interventions based on efforts of community mobilizers and community health workers (CHWs) to reach potential clients and the use of media often play an important role in VMMC demand creation efforts.^8^ Although such strategies are essential and can serve as a catalyst to action, additional interventions are necessary to address the various barriers to male circumcision that have been documented in the literature.^9–11^ Given the influence that one's peers may have on health behaviors such as circumcision uptake,^12^ demand creation strategies that specifically encourage circumcised clients to discuss their experience among their peers have the potential to be effective but have not been piloted and evaluated. Men who have undergone male circumcision may be more effective in promoting uptake among individuals in their social network than community health workers or others who are less strongly connected.^13,14^ They can also directly address barriers such as lack of knowledge and lack of encouragement. In the field of marketing, “viral marketing” is a term that describes the use of existing social networks to raise awareness of products and services and thereby fulfill marketing objectives such as increased product sales.^15^ Understanding ways to promote male circumcision by further leveraging peer effects within social networks can thus be useful for demand creation efforts in Zambia and other countries currently seeking to scale-up VMMC.

This study reports results from implementing an intervention in Zambia that provided small financial incentives to circumcision clients who successfully referred their peers to also seek circumcision.

## METHODS

### Ethical Considerations

The study received ethical approval from the Ministry of Health of the Republic of Zambia, the University of Zambia Biomedical Research Ethics Committee (UNZABREC), and the University of North Carolina at Chapel Hill. We obtained a waiver of consent for the main study because no personal data were collected.

### Study Setting

The study was conducted in the Southern Province of Zambia, where the Centre for Infectious Disease Research in Zambia (CIDRZ) has been providing VMMC services since June 2013. Southern Province is a traditionally noncircumcising region with HIV prevalence of 12.8%. In 2013 and 2014, CIDRZ and 2 other organizations, Society for Family Health (SFH) and Johns Hopkins Program for International Education in Gynecology and Obstetrics, were the main implementing organizations that supported the government's VMMC scale-up in Southern Province.

### Intervention

The intervention allowed clients undergoing male circumcision in intervention facilities to refer up to 5 uncircumcised men in their social network and receive a monetary payment of 10 Zambian Kwacha (US$2) for each person they referred for male circumcision. Men who came as referrals also received 5 peer referral vouchers that they could then provide to uncircumcised men in their social network. The intervention was limited to clients ≥18 years of age.

The amount of US$2 per referral was established after consultation with CIDRZ staff and community members. The amount was also intended to be comparable to payments typically made to mobilizers for VMMC, who earned 500 Kwacha (US$100) per month and contribute to approximately 50 circumcisions per month.

### Intervention and Comparison Facilities

The intervention was implemented at 6 randomly selected CIDRZ-supported facilities in Southern Province where VMMC services were available on at least 1 day each week. Although designed initially as a facility randomized trial, the initial design could not be implemented because of delays in the initiation of VMMC services at several facilities that limited the number of study sites and consequently reduced statistical power. A nonexperimental study design was chosen so that trends in the number of male circumcisions performed at intervention facilities could be compared with trends in 22 nonintervention facilities in Southern Province that were supported by CIDRZ or SFH. Based on 2012 health facility data, the intervention and nonintervention facilities did not have significant differences in various characteristics, including number of beds, number of outreach sites, provision of HIV counseling and testing services, PMTCT services, delivery services, and VMMC services.

### Study Procedures

Before the start of the peer referral intervention, meetings were held in communities served by all the intervention facilities to provide information about the intervention to community leaders and facility staff.

After completion of each circumcision procedure at the intervention facilities, study staff provided peer referral vouchers to VMMC clients ≥18 years of age who were interested in enrolling in the study. Each client received 5 referral vouchers that were valid for a period of 3 months. The vouchers had a unique identification number that allowed the referring person to receive US$2 for each voucher that was subsequently presented by uncircumcised men who came to the facility for VMMC. Each client was also given a card containing the same identification number that was on the referral vouchers. Uncircumcised men who came to the VMMC facilities and underwent circumcision were asked to present the referral voucher to facility staff, who retained the voucher until the referring person came to collect his referral payment. The maximum amount that each client could receive as a result of referring other men was 50 Kwacha (US$10).

At each intervention facility, a log was used to keep track of voucher numbers dispensed and presented. For each client who underwent circumcision, the log contained the name of the client, the date of the circumcision, and the unique identification number on the 5 peer referral vouchers given to the client. Subsequently, each time an individual underwent circumcision and presented one of the referral vouchers, the log recorded the date the voucher was presented and the name of the person who presented it. Clients who referred others for circumcision were then able to claim their referral payment by presenting their card with their unique identification number. Research assistants at each intervention facility who maintained the logs were given the responsibility of disbursing the referral payments.

### Study Duration

The intervention was implemented between mid-June 2014 and mid-February 2015. The intervention period was divided into an active intervention phase of 5 months (4 months in 1 facility where the start of the intervention was delayed) and a passive intervention phase of 3 months. During the active intervention phase from mid-June 2014 to mid-November 2014, referral vouchers were given to all VMMC clients meeting eligibility criteria. During the passive intervention phase, no new referral vouchers were given to VMMC clients, but referral vouchers were collected from clients who had been referred and payments were made to referring persons.

### Data Collection

Data on the number of circumcisions performed each month between January 2013 and February 2015 were obtained from facility registers at each of the 6 intervention facilities and 22 nonintervention facilities. Because several of the facilities did not begin offering VMMC services until well after January 2013, the number of preintervention months of data available from those facilities was reduced.

To learn more about the role of referral vouchers in generating demand for VMMC, research assistants administered a brief follow-up questionnaire by telephone with clients who became circumcised at intervention facilities before the end of the active intervention period. This questionnaire obtained information from clients on demographic and socio-economic characteristics, health behavior, factors that influenced the decision to become circumcised, the role of peer referrals in this decision, and the role of peer referral incentives in clients' efforts to encourage other peers to seek circumcision.

### Statistical Analysis

Using longitudinal data on the monthly number of circumcisions performed in intervention and comparison facilities, the primary analyses used a difference-in-difference model to estimate the likely effect of the intervention on male circumcision uptake. This method, which has been used in the economics and public health literature,^16–18^ effectively compares the trends in monthly circumcisions in intervention and control facilities and tests whether trends in intervention facilities were significantly different during the intervention period. The difference-in-difference model assumes that secular and seasonal trends in all facilities would have been the same had the intervention not been present and therefore includes time-fixed effects to control for these trends. The effect of the peer-referral system intervention is indicated by the coefficient of a binary interaction term indicating whether the month of observation was during the intervention period. In secondary analyses that sought to further understand the role of the intervention in motivating VMMC clients to promote circumcision among their peers, we used data from the follow-up questionnaires and analyzed clients' perceptions of the intervention. To account for the problem of serial correlation within a facility across multiple time periods present in difference-in-difference models,^19^ we used the bootstrap correction proposed by Cameron et al,^20^ specifically designed for a small number of clusters.

## RESULTS

During the 8-month intervention period, 1222 men ≥18 years of age became circumcised in intervention facilities. The majority of circumcisions (N = 848) occurred during the active intervention phase between June 2014 and November 2014 and the remaining circumcisions (N = 374) occurred during the passive intervention phase between mid-December 2014 and mid-February 2015. During the active intervention phase, 699 men were enrolled in the study and received 5 referral vouchers and 348 of these men were referred by men enrolled in the study. An additional 37 men were referred for circumcision during the passive intervention period. Overall, 55% (385/699) of men circumcised during the study period arrived with referral vouchers (Table 1).

**TABLE 1.**
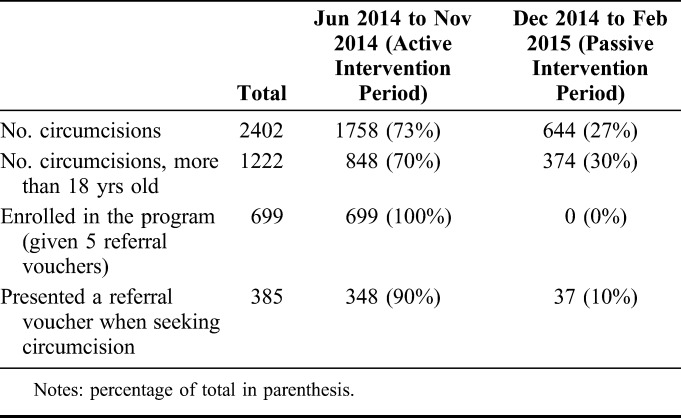
Number of Referrals for Male Circumcision During Intervention Period

**FIGURE 1.**
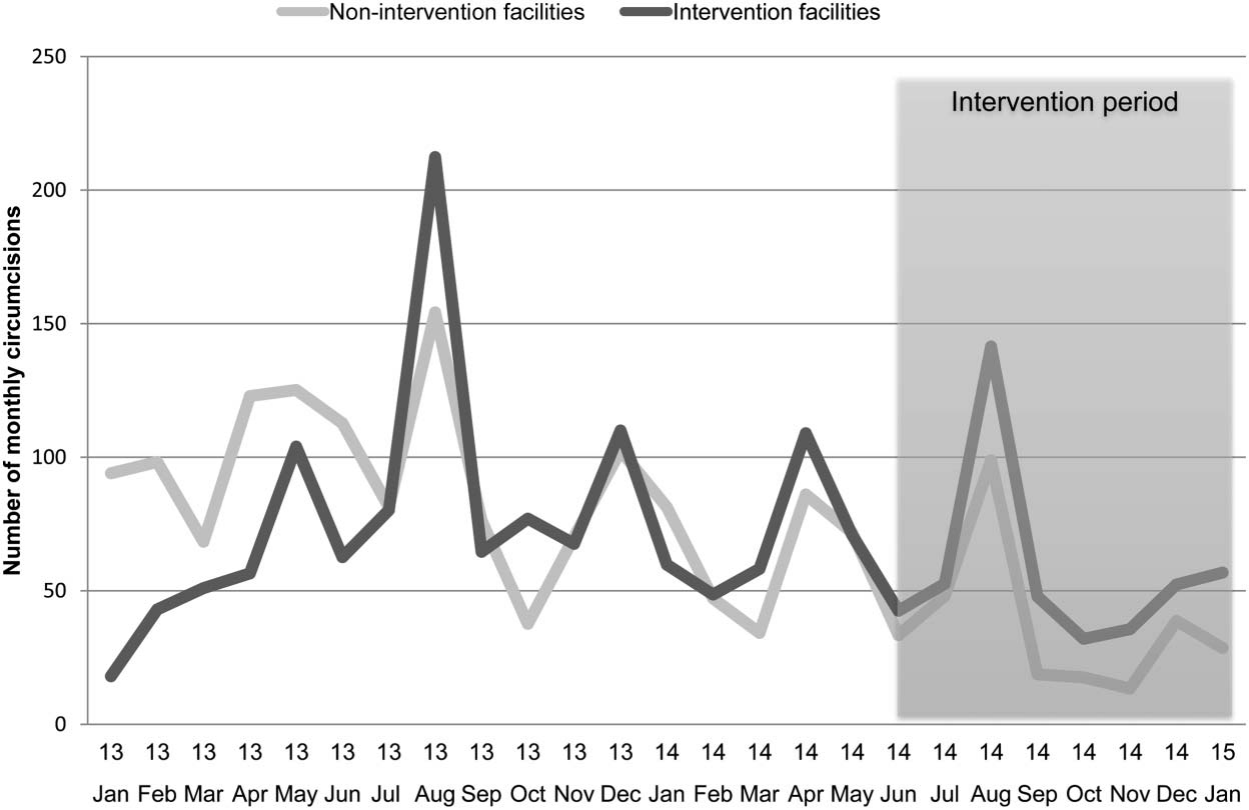
Monthly male circumcisions in intervention and in nonintervention facilities. Notes: graph represents average number of circumcisions per month in intervention facilities (darker line) and nonintervention facilities (lighter line) from January 2013 to January 2015. The shaded area represents the intervention period.

Between January 2013 and February 2015, trends in circumcisions performed at intervention facilities were similar to those in nonintervention comparison facilities supported by either CIDRZ or SFH. During this period, there were 72 circumcisions per month per facility on average, with strong seasonal patterns (Fig. 1). Nearly half (47%) the circumcisions occurred during VMMC campaign months (April, August, and December), when there were intensified efforts to promote male circumcision and services were made more widely available. VMMC demand was also higher during the dry season (April to October) and lower during the rainy season (November to March).

Table 2 reports the results from the difference-in-difference model. The basic model (model 1) uses a binary indicator of whether the facility received the intervention to control for time-invariant differences between all intervention and all comparison facilities, a binary variable to indicate whether a specific month was during the intervention period to control for trends during this period, and an interaction between the 2 variables to test whether the intervention led to a significant difference in the number of circumcisions. The results show that the intervention led to an increase of 7.60 circumcisions per month but that this effect was not statistically significant. The results also reveal a negative trend in average number of circumcisions per month during the intervention period. In a model that instead included month-year fixed effects (binary variables for each month) and facility fixed effects (binary variables for each facility), the effect of the intervention was similar, with a statistically insignificant increase of 10.21 circumcisions per month due to the intervention (model 2). The results were also similar in a separate model that aggregated the set of binary variables for each month into 2 variables only (campaign month or not, and rainy season month or not) (model 3).

**TABLE 2.**
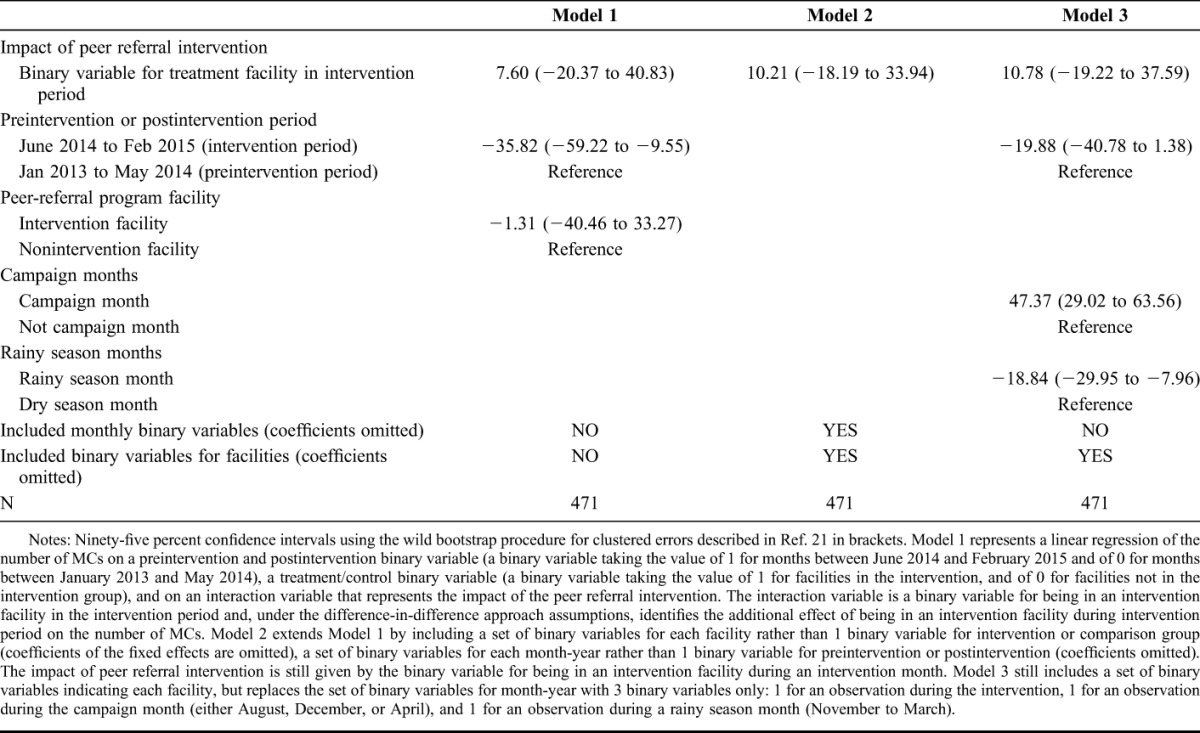
Effect of peer Referral Program on Circumcisions in Intervention Facilities

### Participant Characteristics and Perceptions of the Intervention

The follow-up questionnaire was administered to 289 participants who became circumcised during the active intervention period. The primary reason for not reaching participants was a lack of phone number or network reception. Results from the questionnaire, reported in Table 3, indicate that participants were young (25 years on average), more educated than average given the rural areas (39% had completed upper secondary school or higher) and tended to live near the health facility, with an average one-way travel time of 22 minutes (median 20, IQR 10–30 minutes) and a median transportation cost of 7 Kwacha (US$1.2), (IQR 0–10 Kwacha) to travel to the clinic.

**TABLE 3.**
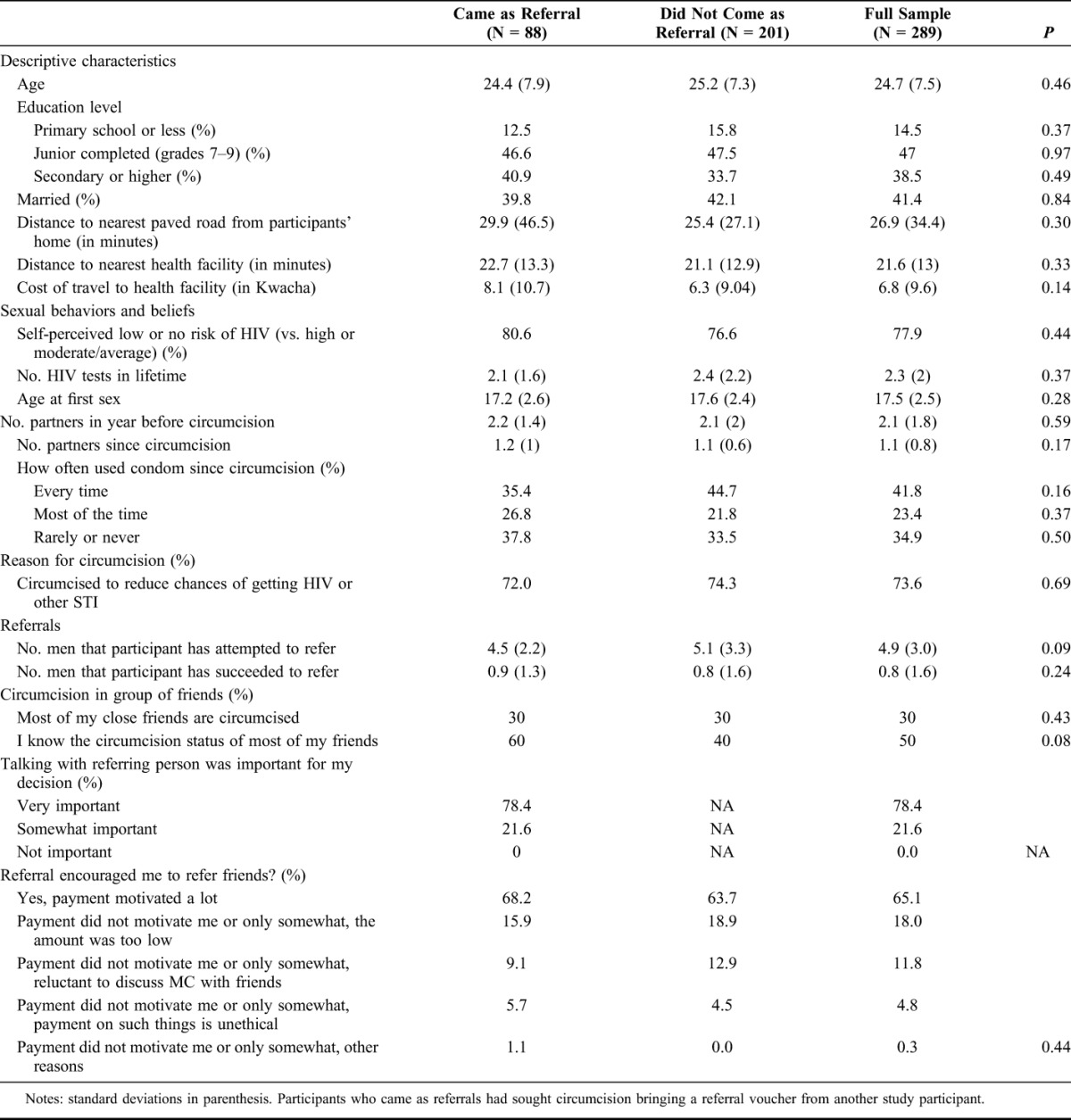
Characteristics of Participants and Perceptions of Intervention

Thirty percent (N = 88) of respondents had been referred for circumcision by other study participants. Respondents reported attempting to refer an average of 5 men, but in practice succeeded in referring an average of 0.8 men. Among men who were referred for circumcision, 78% reported that talking with the person who referred them was “very important” for their decision to seek circumcision. Among all participants, 65% reported that the referral incentive motivated them to refer friends for VMMC “a lot,” and 35% reported that it motivated them “only somewhat” (29%) or “not at all” (6%). Out of the total, 18% reported that the incentive did not motivate them enough because the amount was too low and another 12% because they were reluctant to discuss MC with their friends. Men who attempted referrals and men who did not were no different in terms of age, education, transportation cost, or knowledge of circumcision status of friends.

## DISCUSSION

This study demonstrates the feasibility of an innovative peer referral incentive intervention that sought to encourage men who became circumcised to discuss and promote male circumcision among their friends and relatives. The results from piloting this intervention in 6 facilities offering male circumcision services in the Southern Province of Zambia indicated that many clients did refer at least 1 person and few clients referred multiple persons. After implementation of the 8-month period intervention, a difference-in-difference analysis that compared trends in the number of male circumcisions performed in intervention and nonintervention facilities indicated that the intervention did not result in a significant change in the number of circumcisions.

To our knowledge, this study is one of the first to use peer referral incentives to promote HIV-related health behaviors in sub-Saharan Africa. In other settings, such interventions have been attempted with some success. For example, an intervention in the US that encouraged women to refer their peers for breast cancer screening did generate referrals, although the effect of incentivizing referrals was not found to be statistically significant.^21,22^ Such interventions have also been commonly used in the private sector as part of viral marketing campaigns.^15^ Using peer referral incentive offers several advantages over traditional demand creation approaches that rely on hiring and motivating mobilizers to generate demand. By encouraging each circumcision client to become an advocate for circumcision in their social networks, uncircumcised men who are contemplating circumcision may become more likely to seek VMMC services.

Although the results from this pilot study suggest that the intervention was ineffective in increasing demand for male circumcision, certain features of the intervention and the setting in which it was implemented may have contributed to the lack of effect. An important reason may have been that the amount of peer referral incentive was too small to sufficiently motivate clients to overcome social and cultural barriers to openly discussing male circumcision with their peers and encouraging them to seek VMMC. Because clients who succeeded in referring someone had to return to the health facilities to receive payment, this could have further discouraged peer referrals. In the rural settings where we implemented the intervention, transportation costs are likely to have been an important barrier to the intervention's success and to demand for male circumcision as well. Given the significant HIV prevention benefits of increased demand for male circumcision,^23^ offering larger peer referral incentive amounts may be worthwhile. Taking advantage of novel payment mechanisms such as a mobile phone based money transfer may also facilitate implementation of a peer referral incentive intervention. In addition, improved intervention designs such as a nonlinear incentive structure that offers a larger incentive for each additional referral are also worth consideration.

The limited success of the intervention may also have been because of other barriers to male circumcision that persist. Thus, even if the peer referral incentive intervention was successful in encouraging circumcised clients to *talk* about VMMC with their peers—as data from the questionnaires implemented in this study suggest—other barriers facing uncircumcised men may have limited the effect of the intervention. Given the findings from other studies that provision of a small amount of economic compensation to clients can help overcome barriers to VMMC uptake,^24^ combining peer referral incentives with the provision of direct economic compensation could be a promising demand creation intervention to consider. Other interventions that directly address barriers to VMMC may also be needed alongside future attempts to introduce peer referral incentives.

This study has several limitations. First, the small sample size of facilities in which the intervention was implemented limits the statistical power to estimate its impact on male circumcision demand. Because of factors such as the delayed start of VMMC service provision in the planned number of facilities and logistical challenges in implementing the intervention in many facilities at the same time and over a short period of time, the intervention was only implemented in 6 facilities and efforts were more focused on assessing feasibility and acceptability of incentivizing peer referrals. Additional evaluations of peer referral interventions with cluster randomized designs are needed to establish their effectiveness. This study did demonstrate, however, that clients were willing to refer their peers for VMMC and that payments for such referrals were feasible, although participants may have limited themselves to convincing men who were already contemplating the decision in the immediate future, or whom they would have tried to convince even without the incentive. A second limitation of the study stems from the fact that demand- and supply-side barriers to VMMC—such as large distances between communities and facilities, and service provision on some but not all days of the week—may reduce the generalizability of the results to settings where VMMC is more widely available.

The results from this pilot study suggest that although peer referral incentives for VMMC were feasible and acceptable, offering small incentives for referrals alone was insufficient for increasing demand. However, further efforts to design interventions that encourage users of health services such as VMMC to promote the services among their social networks may yield more promising results. Given the likely influence of peers on individuals' health behavior, identifying more effective ways to encourage interactions between circumcised clients and their peers could hold the key to overcoming barriers to male circumcision and increasing demand.
